# Combining Implicit and Explicit Feature Extraction for Eye Tracking: Attention Classification Using a Heterogeneous Input

**DOI:** 10.3390/s21248205

**Published:** 2021-12-08

**Authors:** Lisa-Marie Vortmann, Felix Putze

**Affiliations:** Cognitive Systems Lab, Department of Mathematics and Computer Science, University of Bremen, 28359 Bremen, Germany; felix.putze@uni-bremen.de

**Keywords:** eye tracking, attention, convolutional neural network, feature extraction, Markov transition fields, Gramian angular fields, heterogeneous feature sets, implicit feature learning

## Abstract

Statistical measurements of eye movement-specific properties, such as fixations, saccades, blinks, or pupil dilation, are frequently utilized as input features for machine learning algorithms applied to eye tracking recordings. These characteristics are intended to be interpretable aspects of eye gazing behavior. However, prior research has demonstrated that when trained on implicit representations of raw eye tracking data, neural networks outperform these traditional techniques. To leverage the strengths and information of both feature sets, we integrated implicit and explicit eye tracking features in one classification approach in this work. A neural network was adapted to process the heterogeneous input and predict the internally and externally directed attention of 154 participants. We compared the accuracies reached by the implicit and combined features for different window lengths and evaluated the approaches in terms of person- and task-independence. The results indicate that combining implicit and explicit feature extraction techniques for eye tracking data improves classification results for attentional state detection significantly. The attentional state was correctly classified during new tasks with an accuracy better than chance, and person-independent classification even outperformed person-dependently trained classifiers for some settings. For future experiments and applications that require eye tracking data classification, we suggest to consider implicit data representation in addition to interpretable explicit features.

## 1. Introduction

Early on, in the beginning of the 20th century, it was discovered that our eyes are a real-time representation of numerous processes occurring in our brain [1]. Whether we are trying to perceive and understand our surroundings, or whether we allow our gaze to wander while our thoughts revolve around something past, eye movements are characteristic of particular mental processes. Obviously of importance are moments when the eyes remain fixed on a point (fixations), movements between fixations (saccades), blinking, or pupil dilation. When it comes to visual perception, our eyes play the most prominent role. In these moments, our attention is directed externally and we attempt to process the sensory input provided by our surroundings. However, even when our attention is directed internally—that is, to thoughts, memories, or ideas—our eyes do not remain stationary [2]. On the contrary, previous research using eye trackers discovered that different mental states result in diverse eye movement patterns [3,4]. Besides attentional states, eye gaze behavior can also be used to classify cognitive or psychological disorders (e.g., autism [5]). Machine learning is utilized to classify these eye movement patterns, and the predictions are employed in a variety of applications [6,7,8,9]. In many circumstances, the eye movements are defined for this purpose with features that quantify the aforementioned fixations, saccades, blinks, and pupil dilations.

In this work, we suggest combining these statistical features that explicitly describe known characteristic eye movement patterns with features that implicitly represent the raw data collected by the eye tracker. While the explicit features are extensively studied and easily interpretable, the implicit features could contain additional information that can be learned by a neural network to complement the presented information. This idea is based on a previous study by Vortmann et al. [10], where the authors compared the classification accuracies using implicit and explicit feature sets. We hypothesize that a heterogeneous feature set that combines the implicit and explicit representations of the eye gaze behavior outperforms the homogeneous feature sets. The classification approach is inspired by feature ensemble learning that has been proven to be very efficient and versatile in a variety of applications, as it deals with multiple classification paths simultaneously [11].

### 1.1. Eye Tracking-Based Attentional State Recognition

The detection of attentional states based on eye tracking data has been at the center of several research works. A major advantage of eye tracking recordings compared to other biosignals (e.g., EEG, fMRI, fNIRS) is the fast and unintrusive setup. Remote eye trackers can be placed in front of a participant or user, and wearable eye trackers allow for flexible recordings outside of standardized laboratory settings. Even webcams can be used to record reliable eye tracking data [12].

Hutt et al. [13] classified mind wandering during lecture viewing significantly better than chance level and discussed the advantages these methods have for massive open online courses. For the classification, they extracted features, such as the number of fixations and the fixation duration, and classified them using Bayesian networks. In [14], Xueling at al. follow a similar motivation and detect internal thought during e-learning. Their features are based on eye vergence information that is available when a binocular eye tracker is used. The obtained results are reliable in times of math-solving, daily computer-based activities (coding, browsing, reading), and free viewing of online lectures.

In this study, our data set will contain periods of internally and externally directed attention. The eye gaze behavior during these attentional states was investigated and analyzed in detail in Benedeck et al. [15]. The authors found significant differences during goal-directed internal attention and external attention. Specifically, internally directed attention was associated with less but longer fixations and blinks and an increased variability of eye vergence and pupil dilation. Additionally, the frequency of microsaccades decreased compared to externally directed attention. In Annerer-Walcher et al. [16], an LSTM was successfully used to classify raw eye tracking signals in times of internally and externally directed attention with an accuracy of 75% on a single trials basis. Another feature representation approach for eye gaze behavior was used in Elbattah et al. [17]. The authors used eye tracking recording to detect autism spectrum disorder by describing the gaze behavior using natural words and text strings (such as fixation and saccade). Methods from natural language processing were then used to successfully classify the data.

### 1.2. Implicit Feature Extraction

The usage of implicit features for classification processes has mainly been studied in the context of text sentiment analysis [18]. In this literature, implicit features are described as features that are not apparent and where the meaning has to be learned. For example, obvious sentiment-describing adjectives are missing, but the structure and other words of a sentence still convey a sentiment that can be learned through machine learning. This idea can be transferred to gaze behavior. While the described eye tracking features represent behavioral patterns that are explicit for eye gaze, there might be hidden information in the time series data of the eye tracker (and in the combination of the eye gaze coordinates and pupil diameter) that is not described by the explicit features or is lost during the quantification of them (e.g., through statistical descriptions). The advantages of implicit feature extraction methods for text-based analyses have been reported in [19,20,21].

Combining machine-learning-based features that were learned from implicit representation of the data and modality-specific features explicitly describing the data into a heterogeneous feature set was also done in previous studies. In the same context of sentiment analysis, Bandana [22] classified the combined feature set using Naive Bayes and support vector machines and claims that the hybrid feature set can be as accurate as other baseline systems.

### 1.3. Implicit vs. Explicit Eye Tracking Features for Attentional State Recognition

In a previous study by Vortmann et al. [10], three different data sets with several tasks and different attentional states were classified. The main goal was to compare classical statistical features of explicit eye gaze descriptions and image representations of the time series data that implicitly describe the data (imaging time series approach, ITS). To generate the implicit images, three different algorithms suggested by Wang and Oates [23] were applied to the data. Wang and Oates [23] showed that the classification of the generated images achieved results competitive with the nine best time series data classification approaches, and the imputation mean square error was significantly reduced compared to using raw time series data. The information contained in the images was extracted and classified by a convolutional neural network, while the explicit features were classified using several state-of-the-art machine learning algorithms. The results showed that the classification accuracies were higher for the implicit features than for the features that explicitly describe the gaze behavior. However, it can be assumed that the explicit features represent different aspects of the data than the images that are used as implicit input features. This additional information could improve the classification further. Thus, in this study, we combine both feature sets.

## 2. Methods

In this work, we will built on the results presented in Vortmann et al. [10] that were presented in Section 1.3. Our main hypothesis is that a combination of the eye tracking-specific features and the imaging time series features as a heterogeneous input for a CNN will improve the classification accuracy for attentional states, compared to homogeneous input features. The results of the two individual feature sets were already compared in Vortmann et al. [10]. They showed that imaging time series features that implicitly describe the eye gaze behavior reached higher classification accuracies for almost all of the performed analyses. Hence, in this study, we will test whether the results achieved with the implicit features can be outperformed using a combined feature set of implicit and explicit features.

As a preliminary analysis, the three different types of images that are generated using the ITS approach will be evaluated further. We will test whether either imaging algorithm alone would be sufficient to reach similar classification accuracies as their combination.

Moreover, the effect of reduced trial lengths on the classification accuracy will be explored. Assuming that the human attentional state switches frequently, shorter time windows would increase the accuracy of real-time predictions. All these aspects will be analyzed for both person-dependent (PD) and person-independent (PI) models. While person-dependent models are highly personalized and often more accurate because they were trained on the data of the person they are also tested on, person-independent models have the advantage that they do not require the previous collection of training data because they are trained on other persons’ data. Real-time classification systems would benefit from person-independent models because the model would work “out of the box” and only require the calibration of the eye tracking device.

Finally, we test the best classification approaches for task independence. The usability of a model highly increases if it can be applied for new tasks without requiring the labeled training data of these tasks.

### 2.1. Data

For the following analyses, we used one of the data sets that was also used in the previous work by Vortmann et al. [10]. The data and the task were originally recorded and presented by Annerer–Walcher et al. [16] and is publicly available. A total of 172 participants performed 16 runs of 6 different tasks (96 trials in total per participant). Of the six tasks, three tasks required internally directed attention, and three tasks required externally directed attention. The three tasks per condition were of different manners: numerical, verbal, and visuospatial. Each task lasted 10–14 s, and the tasks were presented on a computer screen in a randomized order. For a detailed description of the task and the experimental setup, please refer to the original research paper [16].

The gaze direction (X- and Y-coordinates) and the pupil dilation of the participants were recorded using an SMI RED250mobile system (SensoMotoric Instruments, Teltow, Germany) with a temporal resolution of 250 Hz, spatial resolution of 0.03${}^{\circ }$, and gaze position accuracy of 0.4${}^{\circ }$ visual angle.

Incomplete data sets were excluded, which resulted in 154 participants for the following analyses. In Vortmann et al. [10], all trials were cut to a length of 10 s. In this study, we evaluated the classification accuracies for 8 s and 3 s time windows (inspired by the results of Vortmann et al. [24] who found that similar window lengths provided the best trade-off between real-time and classification accuracy). For both versions, the first second of the trial was discarded to exclude the possible contamination of task onset effects. Thus, the 8 s windows contained the recorded eye tracking data from the time interval [1, 9] and the 3 s windows from the time interval [1, 4] after trial onset.

In summary, the position and pupil data of 154 participant were analyzed. Eight- and three-second windows were extracted for all 96 trials of each participant. Half of the trials are labeled as internally directed attention and the other half as externally directed attention; accordingly, the chance level for a correct classification is 0.5.

### 2.2. Feature Extraction

As mentioned before, two different feature extraction approaches were applied. For the first feature set, the generation of features was inspired by eye gaze behavior and characteristic movements. The features are explicitly designed to represent specific attributes extracted from eye tracking data. In contrast, the algorithms used to design the features for the second feature set were designed to visualize time series data in general. Eye movements and pupil dilation are implicitly described through the resulting images that will be used as features.

#### 2.2.1. Explicit Features

Features that can be explicitly designed to describe interpretable eye gaze behavior are often used as the input for classification algorithms. These features are interpretable and based on characteristic movements that have been studied and quantified extensively in past experiments concerning attention. These explicit features include fixations, saccades, and eye blinks [13,15,25,26,27,28,29,30,31,32,33,34], as well as pupil dilation [30,35,36,37,38] and eye vergence [14,15,31,39,40].

The explicit feature set in this study is identical to the statistical summary features of the classical classification approach described in Vortmann et al. [10]. The format of the variables and values recorded by the eye trackers throughout the studies limits which features may be retrieved from the data sets. For the explicit features, we combined fixations, saccades, blinks, residual vergence characteristics, and pupillometric data. The data sequences of X- and Y-coordinates were assessed for fixations, saccades, and blinks in order to extract these characteristics using the PyGaze Toolbox [41]. The blink detection method has a threshold value of 50 ms. Fixations were detected following the dispersion threshold identification algorithm (I-DT) by Salvucci et al. [42] (implementation on github (https://github.com/ecekt/eyegaze, accessed on 2 December 2020)). As proposed by Blignaut [43], the dispersion threshold was set to one degree. The vergence features that could be generated were retrieved using the method outlined in Xuelin Huang et al. [14], and the minimum bounding circles were computed using the nayuki-python project’s script (https://www.nayuki.io/page/smallest-enclosing-circle, accessed on 2 December 2020). We utilized the total value of the computed variable (i.e., number of fixations) as a feature or generated statistical measures to characterize the variable throughout the trial (i.e., mean, standard deviation, median, maximum, minimum, range, kurtosis, and skewness of the distribution of fixation durations). A comprehensive list of all features can be found in Vortmann et al. [10].

#### 2.2.2. Implicit Features

**Figure 1:**
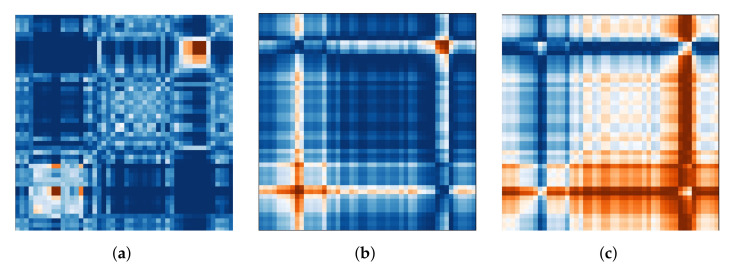
Implicit features: the three images that are generated to represent the X-coordinates of the right eye in one trial with externally directed attention. (**a**) Markov Transition Field (MTF). (**b**) Gramian Angular Summation Field (GASF). (**c**) Gramian Angular Difference Field (GADF).

Three distinct algorithms were used to convert the continuous time series data recorded by the eye tracker into images (see Figure 1). The images are a two-dimensional representation of the time series dynamic through matrices. The matrices can be utilized as input features for the classifier to extract meaningful features implicitly throughout the classification process. These features could represent patterns of eye movement and pupil dilation that are not described by the explicit eye tracking data. Because no information about the X and Y coordinates is available, periods with identifiable blinks were filtered from the data in the first stage. A full discussion of the approaches may be found in Wang and Oates [23].

We chose to generate images for each eye independently, with one image representing the X coordinate, one representing the Y coordinate, and one representing the current pupil diameter recorded by the eye tracker. This keeps us as near to displaying the raw data as possible while still giving the neural net the ability to identify and learn from differences and similarities between the eyes (following the notion of utilizing vergence characteristics). The first transformation algorithm is the Markov transition field (MTF), which creates a matrix based on transition probabilities. The data sequence is divided into quantiles based on the magnitude of the values. Each data point is allocated to a quantile, and a weighted adjacency matrix is built by counting the transitions from sample to sample between quantiles along the time axis using a first-order Markov chain. This Markov transition matrix is then normalized and spread out over the magnitude axis while taking into account the temporal locations, yielding the MTF. The main diagonal depicts the likelihood of self-transition at each time step. Figure 1a shows an example image generated for the X coordinates of the right eye in one trial with externally directed attention.

Additionally, we use two variants of the Gramian angular field transformation algorithm. The first is known as the Gramian angular summation field (GASF, see Figure 1b) and the second as the Gramian angular difference field (GADF, see Figure 1c). The data sequence is rescaled to [−1, 1] and then represented in polar coordinates by encoding the data values as the angular cosine and the corresponding timestamp as the radius in both approaches. As a result, the data series is moved from the Cartesian coordinate system to the polar coordinate system, which has the advantage of preserving the absolute temporal relationship for all points. To determine the temporal correlation within time intervals, we calculate the trigonometric sum or difference pairwise between the points. As a result, the Gramian matrix has the dimensions n×n, where *n* is the length of the raw time series. The trigonometric difference/sum with regard to the time interval is represented by each cell. Each cell on the main diagonal has the original value/angular information and can be used to reconstruct the original time series.

Piecewise aggregation approximation (PAA) can be used for blurring to reduce the size of the output images [44]. The effect of blurring was discussed in Vortmann et al. [10].

The pyts-toolbox for python was used to turn the data sequences into the MTF, GASF, and GADF images [45]. The image size was set to 48 × 48 pixels, and all pixel values for individual images were normalized between [−1, 1]. Following this, all generated images (3 transformations * 2 eyes * (X/Y-coordinates + pupil diameter) = 18 images) were concatenated into a 3 × 6 image matrix. As mentioned before, this image generating approach was used on valid (non-blink) data from single trials per condition [46].

### 2.3. Classifier

As the classification algorithm, we chose an adapted version of the SimpleNet in Vortmann et al. [10] that was designed using the suggestions of Yang et al. [47]. In Vortmann et al. [10], it was shown that the classification accuracies achieved by this convolutional neural network (CNN) using the implicit ITS features were state-of-the-art results. To suit the current study, the CNN was adjusted to be trained on the heterogeneous input features. The first two layers are convolutional layers with a kernel size of 5×5 processing only the implicit images of the trial. After the second convolution, a two-dimensional max pooling is applied to the signal. Next, a dropout layer [48] was included that temporarily zeros out two-dimensional channel data with a probability of 0.5 to avoid overfitting, followed by a fully connected layer. Thus far, only the implicit features were processed in the CNN. This first stage allows for the network to learn important features from the images that implicitly describe important eye gaze behavior for the discrimination between the attentional states. After the first linear layer, the explicit features that statistically describe the classical eye movement characteristics are optionally added (only during the classification of the combined feature set). After applying a batch normalization [49] with a momentum of 0.1 to the explicit features, the two preprocessed feature sets are internally concatenated. The signal is passed through two linear layers, another dropout layer, and two final linear layers. The number of units of the output layer is identical to the number of possible classification labels (in our cases: 2). The layers of the network, the output shapes, and the number of learnable parametery for each layer are described in Table 1. As can be seen in the table, the number of parameters changes slightly if the explicit features are added (combined feature set). In the first stage, before adding the explicit features, a rectified linear unit (ReLU) was used as the activation function after each layer. In the second part of the CNN, the hyperbolic tangent (tanh) was used. These settings proved to reach the highest classification accuracies for our data. The neural net was implemented using PyTorch for Python [50].

## 3. Results

The comparison of implicit and explicit eye tracking features for the classification of attentional states from this data set was already discussed in Vortmann et al. [10] and showed that our ITS features that implicitly describe the eye gaze in times of internally or externally directed attention outperformed explicit, statistical features describing common eye movement characteristics. We will first report a comparison of the three algorithms that were used for the image generation in the implicit feature set. Secondly, the classification accuracies using a homogeneous feature set of only the ITS features and a heterogeneous feature set that combines the ITS and the classical eye tracking features will be analyzed. Afterward, the results of a person-independent classifier will be presented, as well as the analysis of task independence. In a final step, the differences between the 8 and 3 s windows for all aforementioned comparisons will be analyzed for significance.

For all participant-dependent results, the average fold accuracy of a five-fold cross-validation with a shuffled, stratified split will be reported. For the person-independent results, the classifier was trained in a leave-one-out fashion. For each of the 154 available full data sets, the classifier was trained on 153 data sets and tested on the left out 154th data set. Further methodological details will be explained in the following.

The achieved accuracies will be evaluated based on the suggestion by Müller-Putz et al. [51] to calculate a threshold above which the classifier performs better than a random one with a confidence of 95%. All statistical analysis will be corrected for multiple testing assuming a false detection rate (FDR) of α=0.05 following Benjamini and Hochberg [52]. For all statistical tests, a significance threshold of 0.05 will be assumed.

### 3.1. Analysis of Implicit Features

**Figure 2:**
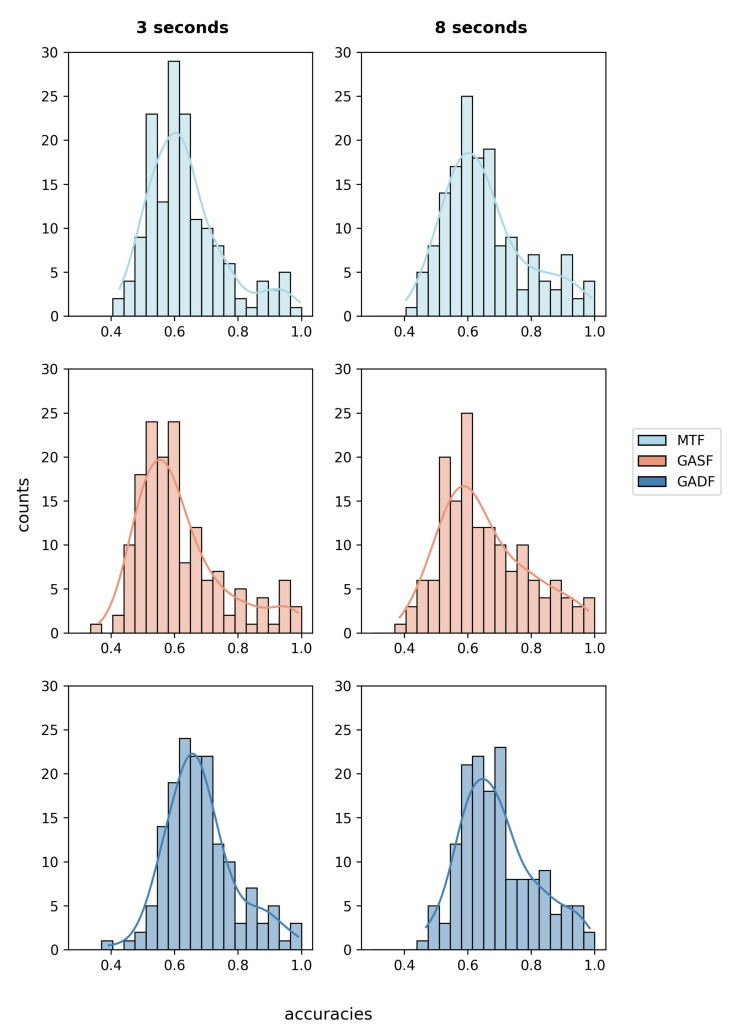
Average fold accuracy per participant represented as histograms. As input features, only the features that were generated using either of the presented algorithms were used. The histograms on the left show the results for the 3 s windows, while the histograms on the right represent the 8 s windows.

The ITS features that implicitly represent the X- and Y-coordinates and the pupil diameter of both eyes were generated using three different algorithms: Markov transition fields, Gramian angular summation fields, and Gramian angular difference fields. In the first step, we analyzed if either of the three image generating algorithms alone reaches classification accuracies better than chance for the majority of participants. The described CNN was trained and tested per person in a five-fold cross-validation using only the images generated using each of the algorithms alone. Hence, an image matrix consisting of six images (three for each eye) was used as the input feature set. Figure 2 shows the distribution of achieved average fold accuracies for each algorithm on the 3 and 8 s windows.

For the 3 s windows, the lowest average accuracy across participants of 0.616±0.135 was obtained using only the GASF features. The classifier only performed better than a random one [51] for 27.3% of the data sets. Using only the MTF features improved the average accuracy to 0.636±0.12, and the best results were achieved using only the images that were calculated using the GADF algorithm. The mean accuracy reached 0.682±0.11. The 95%-confidence interval for a classification using only GADF features is [0.664, 0.699], and the classifier was significantly better than a random one [51] for more than half of the data sets.

Compared to the CNN that is trained on the 18 images of all algorithms (M = 0.687, SD = 0.115), the MTF- and the GASF-based classifiers perform significantly worse (MTF: t(154)=−7.63,p<0.001; GASF: t(154)=−12.44,p<0.001). The results of the GADF-based classification for 3 s data windows do not differ significantly from the results of a classifier trained on all images, t(154)=−0.806,p=0.4215.

Similarly, for the 8 s windows, the best individual results are achieved using only the GADF features. An average accuracy of 0.694±0.12 with a 95%-confidence interval between [0.676, 0.713] and 53.1% of data sets better than random [51] are achieved. In comparison, the MTF-based classifier had a mean accuracy of 0.655±0.13 and the GASF classifier of 0.654±0.138.

All of the single-algorithm classifiers for the 8 s windows perform worse than a classifier trained on their combination (M = 0.714, SD = 0.13). The differences are highly significant for the MTF features (t(154)=−9.122018, p<0.001) and the GASF features (t(154)=−8.411133p<0.001) and significant for the GADF features (t(154)=−3.351624, p=0.001).

Despite there being no significant accuracy difference for classifiers trained on only GADF features and all three algorithms for 3 s data windows, we will perform all following studies using the combined image matrix of the MTF, GASF, and GADF as input features. The best results were achieved using all 18 images. As the main goal of the study is the exploration of a heterogeneous feature set of implicit and explicit eye tracking generation for attentional state classification, we want to use optimized feature sets. A reduction of the number of images would reduce the computation time, but this will be evaluated further in future work.

### 3.2. Comparison of Homogeneous and Heterogeneous Feature Sets

The main hypothesis of this research is the superiority of a heterogeneous feature set that combines implicit and explicit eye tracking features for attentional state classification compared to a homogeneous feature set containing either implicitly generated or explicitly designed features. As a first within-person analysis, we trained and tested the described CNN in a five-fold cross-validation either on the ITS image matrix only or added the explicit features for a combined feature set. This was done for 3 s and 8 s data windows. The statistical values describing the average fold accuracies across participants can be seen in Table 2.

**Figure 3:**
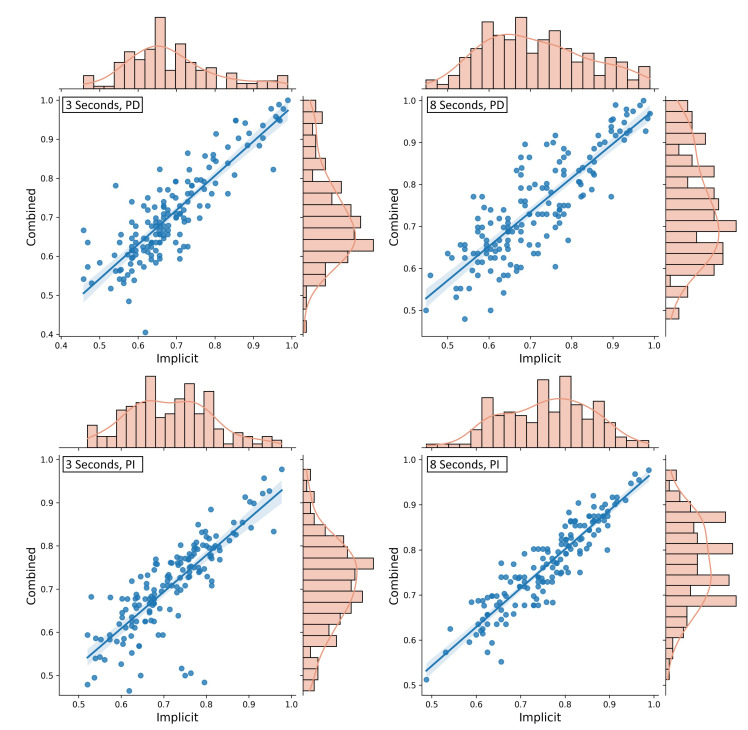
The distributions of implicit and combined feature sets represented as histograms and scatter plots including a visualization of the correlation. The 3 s windows are displayed on the left, and the 8 s windows are displayed on the right. PD = Person-dependent; PI = Person-independent.

For 3 s windows of eye tracking data, the classification accuracy on combined features (M = 0.706, SD = 0.118) improved the results of 57.8% of the participants compared to only the implicit features (M = 0.687, SD = 0.115). The difference between these two approaches is highly significant (t(154)=−3.976,p<0.001). An analysis of the correlation between the classification results using the Pearson correlation yields a strong positive correlation of r=0.86 (see Figure 3, upper left).

For the 8 s windows, an accuracy improvement using the combined feature set (M = 0.745, SD = 0.122) in comparison to the implicit features only (M = 0.714, SD = 0.125) was found for 66.2% of the participants. Again, the difference between the two approaches is highly significant with t(154)=−5.499, p<0.001, and the accuracies are strongly correlated with r=0.837. The exact distribution can be seen in Figure 3 (upper right).

In summary, the CNN that was trained and tested on the combined feature set outperformed a CNN with the homogeneous input features for both window lengths. This supports our main hypothesis and suggests including implicit eye tracking features during the classification process for attentional states. In a next step, the results of this person-dependent classification approach will be tested for person and task generalizability. A classifier that is trained person-independently and used to label previously unseen participants would exclude the need for person-specific training data. This would highly increase the usability of such a classification system.

### 3.3. Person-Independent Classification

To evaluate the results that are achieved when the CNN is trained and tested across participants, we chose a leave-one-participant-out approach for training and testing. Hence, the classifier was always trained on all but one data set and tested on the data set of the remaining participant. Following the main research question of the study, we compare the results of a combined feature set to the results of implicit input features only.

Using 3 s data windows and the homogeneous feature set containing the implicit image features, an average accuracy of 0.717±0.098 was achieved. The data sets of 90.91% of the participants could be classified with an accuracy better than random [51]. With a confidence of 95%, the accuracy was within the interval [0.701, 0.732]. These results were better than the results when the explicit features were added to the feature set (M = 0.708, SD = 0.103) where the 95%-confidence interval was [0.691, 0.724]. For the combined feature set, only 83.12% of the data sets were classified with an accuracy better than random [51]. However, the difference is not significant, t(154)=1.768,p=0.079. Using the combined feature set improved the classification results for 43.51% of the participants. The accuracies of both approaches were strongly correlated with a Pearson’s correlation of r=0.805 (see Figure 3, lower left).

The accuracy distributions using the implicit and combined feature sets on 8 s windows of eye tracking data can be seen in Figure 3 (lower right). For the classification on the homogeneous feature set of only the image features (M = 0.76, SD = 0.1), the 95%-confidence interval was [0.744, 0.776]. For the classification on the heterogeneous feature set of implicit and explicit features (M = 0.767, SD = 0.094), the 95%-confidence interval was [0.752, 0.782]. Both approaches classified 96% of the data sets with an accuracy better than random classification [51], but the combined feature set improved the results for 50.65% of the participants. The difference between the two feature sets was modest but significant with t(154)=−2.15,p=0.033, and the correlation was strong with r=0.916.

Using a leave-one-participant-out cross-validation, the advantages of a combination of implicitly and explicitly designed features are not as strong as for person-dependently trained models. Strikingly, as already found in Vortmann et al. [10], the results using the person-independently trained CNN are more accurate than for within-participant training and testing.

**Figure 4:**
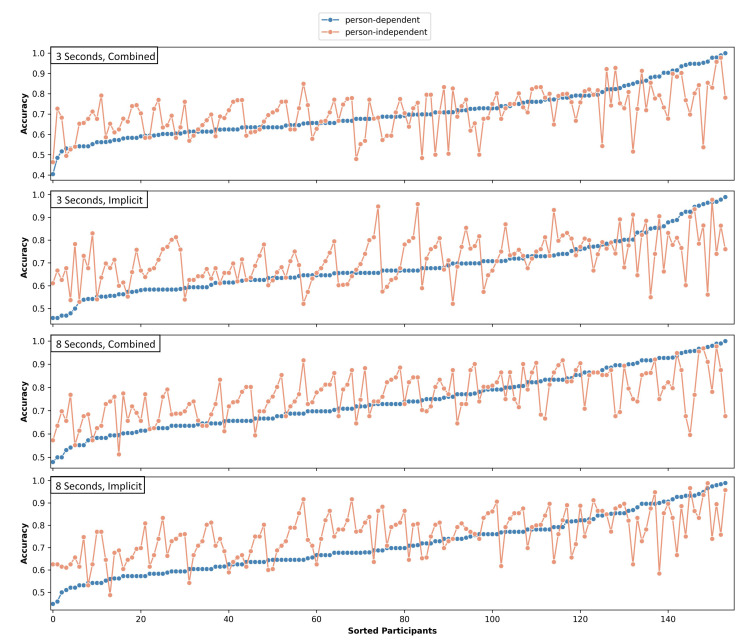
Person-dependent and person-independent classification accuracies per participant. Each plot is sorted individually by ascending person-dependent classification accuracy.

The classification accuracy using only the implicit image features generated on 3 s data improved for 62.99% of the data sets when the person-independent classifier was applied. This improvement was mainly present for participants with a mediocre person-dependent classification accuracy. While the number of data sets exceeding 75% accuracy raised from 36 to 57, the number of data sets above a threshold of 90% accuracy dropped from 11 to 8 using the independently trained CNN. The accuracy difference is significant (t(154)=−3.287, p=0.0013), and the results are moderately positively correlated (r=0.44). Figure 4 shows the independent and dependent results per participant ordered by ascending person-dependent classification accuracies.

Similar effects can be seen for the combined feature set computed on the 3 s windows. The classification accuracy of 55.2% of the participants was higher for an independently trained network compared to person-dependent training (see Figure 4). The results were moderately correlated (r=0.518), but the differences were not significant (t(154)=−0.152,p=0.88).

On the 8 s data windows, an improvement was achieved for 70.13% of the data sets using the person-independent combined feature set compared to the ITS image-based classification. Again, mediocre accuracies were raised; the number of participants with an accuracy above 75% increased from 58 to 82, whereas the number of participants with an accuracy above 90% dropped from 15 to 9. The difference was highly significant with t(154)=−5.315,p<0.001, and the results were again moderately correlated with r=0.566 (see Figure 4).

If the combined heterogeneous feature sets were used for 8 s windows, the independent classifier outperformed the dependent classifier on 59.74% of the data sets. Figure 4 shows the ordered dependent and independent results per participant. The difference was significant (t(154)=−2.564,p=0.011), and the results are moderately correlated (r=0.559).

Except for the combined feature set of the 3 s windows, the person-independent classification was significantly better than the person-dependent classification. The main improvements were observed for lower and mediocre person-dependent classification accuracies. Data sets with a high person-dependent accuracy usually had a slightly decreased person-independent accuracy.

### 3.4. Task Independence

As in the original paper by Annerer-Walcher et al. [16], we tested the task generalizability of a classifier trained on the heterogeneous feature set. A classifier that can label data from previously unseen tasks has a higher usability and is highly desirable. In this analysis, the training set was made up of four of the six tasks, two internal and two external. The remaining internal and external tasks were used as the test data. This was done for all nine possible combinations of internal and external tasks. The data sets of all participants were combined in this analysis. We report the average classification accuracy of the nine classifications using the combined feature set.

For the 3 s data windows, the average classification accuracy reached 0.587±0.027. Despite the lowest accuracy of 0.53, all of the combinations led to classification accuracies better than random (following Müller-Putz et al. [51]). With a confidence of 95%, a data set combined of previously unseen internal and external tasks will be classified correctly between [0.566, 0.61].

The accuracy increased to 0.624±0.03 for the 8 s windows, and all combinations were classified with an accuracy between 0.591 and 0.676.

### 3.5. Trial Length Comparison

All analyses in this study were performed on 3 s and 8 s windows. Lastly, we will compare the classification accuracies achieved on both of these settings for significant differences.

**Figure 5:**
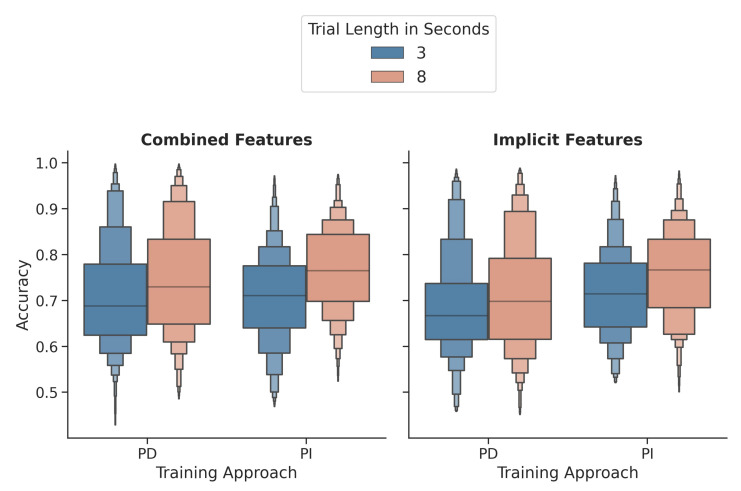
Comparison of the 3 and 8 s windows for person-dependent and independent classifiers using the combined and the implicit feature sets. PD = Person-dependent; PI = Person-independent.

Figure 5 shows the distribution for person-dependent and independent classifiers of both window lengths using the combined and the implicit feature sets. For the implicit image features, the differences between the 3 s and 8 s windows were highly significant for person-dependent (t(154)=−4.096,p<0.001) and person-independent (t(154)=−8.068,p<0.001) classification. The 8 s windows were always classified with a higher accuracy on average. The same is true for the combined features with t(154)=−5.802,p<0.001 for the person-dependent classifier and t(154)=−9.156, p<0.001 for the independent-classifier.

Looking at the task-independently trained and tested classifiers, the differences between both window lengths are also significant (t(9)=−3.343,p=0.01). Only for the comparison of the individual image generation algorithms, not all differences were significant. While the MTF and GASF on 3 and 8 s windows resulted in significantly different accuracies (MTF: t(154)=−2.767,p=0.006; GASF: t(154)=−5.75,p<0.001), the GADF results did not differ (t(154)=−1.91,p=0.058).

Overall, the results using 8 s eye tracking windows were higher compared to the 3 s windows for all analyses except for the classification on GADF-generated images only.

### 3.6. Summary

The best results were achieved using a person-independently trained classifier of the heterogeneous feature set that combines implicit and explicit features generated for 8 s eye tracking windows. The average accuracy for all 154 participants was 76.7%. In summary, we found that

- A feature set that only contains MTF, GASF, or GADF images results in a decreased accuracy compared to all three algorithms.
- A combined feature set outperforms a homogeneous feature set.
- Person-independent classification improves the results for participants with low or mediocre person-dependent classification results.
- On average, the person-independent classifiers outperformed the person-dependent classifiers.
- Task-independent classification is possible with an accuracy above chance.
- Eight-second data windows result in a higher classification accuracy than 3 s data windows for almost all settings.
- Both 3 s and 8 s data windows allow for reliable classification using person-dependent or person-independent classifiers.

## 4. Discussion

In the presented study, we evaluated the hypothesis that the combination of explicit eye tracking features with implicitly designed features that describe the raw time series data improves the classification accuracy for attentional states. Implicit features could contain information that was lost during the extraction of the explicit features. In Vortmann et al. [10], it was shown that an implicit feature set of images generated using the imaging time series approach by Wang et al. [23] resulted in higher classification accuracies than explicitly extracted eye tracking-specific features that are used in state-of-the-art approaches. We used a convolutional neural network for the classification of either the homogeneous implicit feature set or a heterogeneous combined feature set. The hypothesis was tested for person-dependent and person-independent classifiers, 3 s and 8 s windows, and task independence. Our results on a data set of 154 participants collected by Annerer-Walcher et al. [16] proved that a combination of implicit and explicit features further improves the achievable classification accuracies.

A comparison of the results from this study with the results in Vortmann et al. [10] is only possible to a limited extent because some settings of the feature generation and the CNN were altered. While the results in the previous paper were only reported for 10 s windows, we report 8 s and 3 s windows in this work. Furthermore, the ITS images were only created for the left and right eye’s X- and Y-coordinates. We have included images obtained for the pupil diameter of both eyes in the presented study.

Overall, the main hypothesis was proven to be correct for this eye tracking data set. A heterogeneous feature set of explicitly designed and implicitly describing features improves the achievable classification accuracies compared to homogeneous feature sets of either modality.

### 4.1. Implicit Features

The feature generation algorithms used in this and the previous work are only one option to implicitly represent the eye tracking recordings. Other time series representations can be chosen as input features for the classification of attentional states based on eye tracking data (e.g., scanpath images, raw time series data). Future work will explore other algorithms.

We generated images from the recorded time series as implicit features. The three approaches (MTF, GASF, GADF) each depict a distinct component of the data that may not be reflected in the explicitly extracted statistical features that define fixations, saccades, blinks, and other eye tracking-specific properties. Certain patterns of eye movement or pupil diameter variations, for example, may be portrayed in images that are lost during classical feature generation. The analysis of classification accuracies on single algorithm feature sets showed that their combination increases the preciseness of the predictions for attentional states. We found that the best results can be achieved using the GADF algorithm. However, for 8 s windows, the combination of all three image generation algorithms is still significantly better. Reducing the amount of features would be beneficial in terms of reducing computation time and memory requirements, but there appears to be a trade-off because it degrades the results. A combination of two of the three algorithms is needed to identify the optimal feature set for the implicit features. Moreover, the significance of each time series (X- and Y-coordinates, pupil diameter) should be evaluated, and the impacts of simply utilizing the right or left eye should be investigated. The focus on high accuracies or low computing times can be altered depending on the context in which the classifier will be used, and the feature set should be chosen accordingly.

### 4.2. Window Length

The same philosophy holds for the chosen window length. In this study, we compared 8 and 3 s windows. While the 8 s windows resulted in significantly higher classification accuracies on average, 3 s windows might be beneficial for certain classification contexts. For tasks and applications in which a frequent switch of attentional states can be expected, shorter data windows are required for precise labeling. In general, the optimal window length is highly dependent on the study goal. For online real-time classification systems, shorter shifting windows should be considered, while the offline analysis of more controlled experiment settings can be performed on longer windows. Another aspect that has to be considered for the real-time application of the results is the computational time that is needed to generate the images and perform the classification. Future work should explore this direction more. For the representation of the data as images using the MFT, GADF, and GASF algorithms, the sampling rate of the data and the resolution of the images also need to be considered. In this work, 3 s and 8 s windows were both represented as 48 × 48 pixel images. This means that the data of the 8 s windows are downsampled to a higher extent in the representation than the 3 s windows.

In the future, a more detailed analysis of the interplay of sampling rate, window length, and image resolution should be performed. An important aspect to keep in mind is that the appropriate representation of the data could also depend on the attentional states that are to be classified.

Compared to the implicit image results for the 10 s windows in Vortmann et al. [10], the results using 8 s windows were better for person-dependent and person-independent classifiers. However, as mentioned before, these improvements might be due to the adjusted settings.

### 4.3. Classification Approaches

We trained and tested the CNN using two different approaches: person-dependent and person-independent. Usually, due to inter-participant differences, person-dependently trained classifiers are expected to achieve higher classification accuracies. However, the available training data for the person-independent classifier is much higher. A larger variance in the training data can reduce the bias in the classifier and enhance the label prediction on unseen data. In our results, the average preciseness of the classification was higher using person-independent classifiers. Looking at the results per participant, it was shown that the improvement is mainly present for data sets where the person-dependent classification accuracy was lower. Especially for participants with a person-dependent classification accuracy of above 90%, the person-independent classifier decreased the results. The classification approach used in future tests is determined by the quantity of accessible data sets and the desired application. The most significant advantage of person-independently trained classifiers is that they do not necessitate the acquisition of training data for each participant. This means that a classification may be conducted “out-of-the-box” in a real-time online system. The utility of such a system would be greatly enhanced if person-independent classification was reliably possible. However, training would very certainly require a large training data set that would need to be recorded beforehand.

The strong decrease of accuracy for some participants when an independent classifier is used compared to personalized classification is possibly due to the inter-person differences than can be observed in most eye tracking experiments. The precise nature of these fluctuations and which features are most valuable for both classification approaches could be studied further in future experiments. In general, a thorough examination of the feature importances would be really beneficial. On the one hand, classification accuracies for all approaches might be compared using only subsets of the features in order to discover the most valuable features. Explainable AI methods, on the other hand, may be used to comprehend the weights and parameters learned by the CNN for both classification approaches. An analysis of relevant patterns in the implicit image features can shine a light on the additional information included in the implicit but not the explicit features.

It can be observed in this study that the improvement for the heterogeneous feature set compared to the the implicit feature set is not as high for person-independent classification as it is for person-dependent classification. This hints at the higher importance of the explicit features during the person-independent classification. This is in accordance with the findings of Vortmann et al. [10], where the person-independent classification based on implicit features (M = 0.743) outperformed the explicit features (M = 0.689) for this data set. Thus, in this study, adding the explicit features does not lead to a significant increase in classification accuracy for 3 s windows and only to a slight increase for 8 s windows (increase of 0.7% on average).

To bridge the gap between person-dependent and person-independent classifiers, the next step would include transfer learning approaches. A pretrained classifier on person-independent data could be retrained using person-specific data to increase the accuracy for each participant further. The usefulness of such an approach is, once again, strongly reliant on the context in which the classifier is to be used.

The convolutional neural network that was used in this study was chosen because the implicit features are represented by images. CNNs are very successful in image classification tasks [53]. However, the CNN could be optimized further, and other deep learning approaches could be tested to classify the feature sets. Alongside the exploration of other implicit data representations, as suggested before, other classification algorithms might further improve the achievable accuracies.

### 4.4. Generalizability of the Results

A first attempt at evaluating the generalizability of the results was performed through task-independent training and testing. First, it needs to be noted that the results cannot be compared to the results of Vortmann et al. [10] because the splitting into test and training data was different. The previous study only used one of the tasks as the test set, while this study used all possible combinations of an internal and an external task as the test set. This approach was also used in Annerer-Walcher et al. [16], and they report an average accuracy of 61.1% using the LSTM as reported before. Our results on the 8 s data windows (M = 62.4%) outperform the classifier in terms of task independence. For future studies, additional data sets need to be evaluated to reproduce the results. As mentioned before, this includes other sampling rates for the data and also other available time series combinations provided by eye trackers.

The comparisons performed in Vortmann et al. [10] suggested that the implicit features work well for internally and externally directed attention discrimination. However, the results for the classification of attention on real and virtual objects was not possible for person-independent classifiers based on the implicit features. The generalizability of our results in this study to other attentional states needs to be tested in the future.

Concerning the generalizability of the results to new participants, we are confident that the reported results are representative for new recordings. The high number of participants and performed cross-validations reduce a possible bias in the data. This is also visible in the calculated 95%-confidence intervals that usually span a rather narrow frame for expectable classification accuracies. For example, with a certainty of 95%, a participant’s data will be classified with an accuracy between 0.744 and 0.776 if the person-independent 8 s classifier with the heterogeneous feature set is used.

### 4.5. Contribution to the Field

Following the findings of this work, we propose that during the classification of eye tracking data, implicit data representations should be used as input characteristics in addition to explicitly designed features. While this study was conducted on a data set that included different attentional states, it is plausible to infer that the findings may be applied to other mental states as well. We investigated person-dependence and person-independence, as well as different window lengths, and discussed the results in the context of various applications or use cases.

To summarize, while the decisions on window length, training approach, and feature choice are context dependent, the combination of implicit and explicit features is always advisable.

## Figures and Tables

**Table 1:** Layer types, output shapes, and the number of parameters for the CNN that was used to classify the data. For the combined feature sets, the explicit features were added intermediately at the indicated layer.

Layer (Type)	Output Shape		Param #	
Conv2d	[−1, 60, 44, 44]		27,060	
Conv2d	[−1, 120, 18, 18]		180,120	
Dropout2d	[−1, 120, 18, 18]		0	
Linear	[−1, 500]		4,860,500	
*Combined feature set?*	*No*	*Yes*	*N*	*Yes*
BatchNorm1d		[−1, 75]		150
Linear	[−1, 300]	[−1, 300]	150,300	172,800
Linear	[−1, 120]	[−1, 120]	36,120	36,120
Dropout	[−1, 120]	[−1, 120]	0	0
Linear	[−1, 20]	[−1, 20]	2420	2420
Linear	[−1, 2]	[−1, 2]	42	42
**Total**			5,256,562	5,279,212

**Table 2:** Statistical values representing the classification results of all 154 participants for 3 and 8 s windows to compare the implicit and combined feature sets.

		Feature Set	
Window Length	Statistic	Implicit	Combined
	Mean	0.687	0.706
	Standard deviation	0.115	0.118
	Minimum	0.458	0.405
	Maximum	0.99	1.0
3 s	95%-Confidence Interval	[0.668, 0.705]	[0.688, 0.725]
	Better than chance (absolute)	79	89
	Better than chance (relative)	0.513	0.578
	Above 75% accuracy	36	47
	Above 90% accuracy	11	15
	Mean	0.714	0.745
	Standard deviation	0.125	0.122
	Minimum	0.448	0.48
	Maximum	0.99	1.0
8 s	95%-Confidence Interval	[0.693, 0.734]	[0.725, 0.764]
	Better than chance(absolute)	94	108
	Better than chance (relative)	0.61	0.701
	Above 75% accuracy	58	65
	Above 90% accuracy	15	22

## Data Availability

The analyzed data set is available at https://osf.io/scmry/ (accessed on 2 July 2021).

## Abbreviations

The following abbreviations are used in this manuscript:

ITS	Imaging time series
CNN	Convolutional neural network
PD	Person-dependent
PI	Person-independent
MTF	Markov transition field
GASF	Gramian angular summation field
GADF	Gramian angular difference field
ReLU	Rectified linear unit
tanh	Hyperbolic tangent
FDR	False detection rate
